# Supplementary material for: Comparison of two IGRA assays exploring cell-mediated immunity against CMV, BKV, and EBV in kidney transplant patients
Source: Microbiol Spectr. 2026 Feb 27;14(4):e02289-25. doi: 10.1128/spectrum.02289-25 (PMC13055251; doi:10.1128/spectrum.02289-25)

# **Supplementary data**

Supplemental Figure 1: Workflow of the study


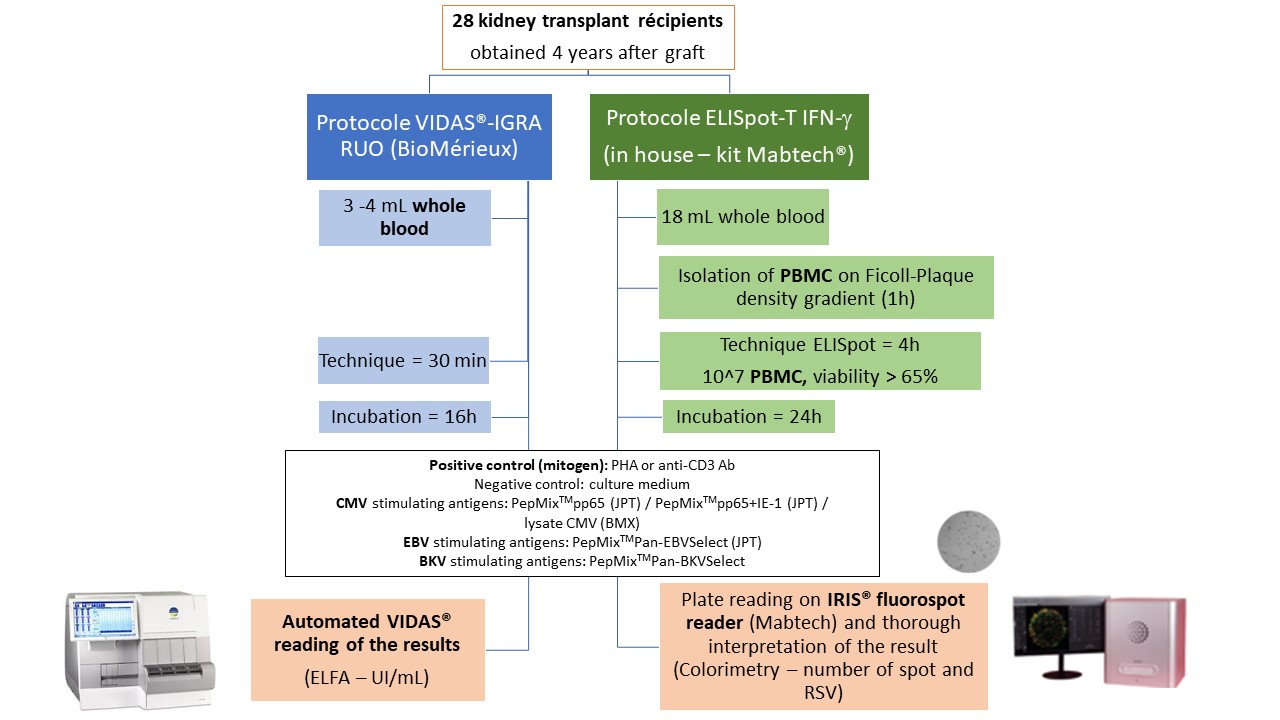


*PBMC: peripheral blood mononuclear cell; PHA: phytohemagglutinine; ELFA: Enzyme-linked fluorescent assay, JPT: JPT Peptide Technologies GmbH*

Supplemental Table 1: Agreement between ELISpot and VIDAS^®^-IGRA quantitative results obtained after activation of T-cells with a) pp65; b) CMV lysate.


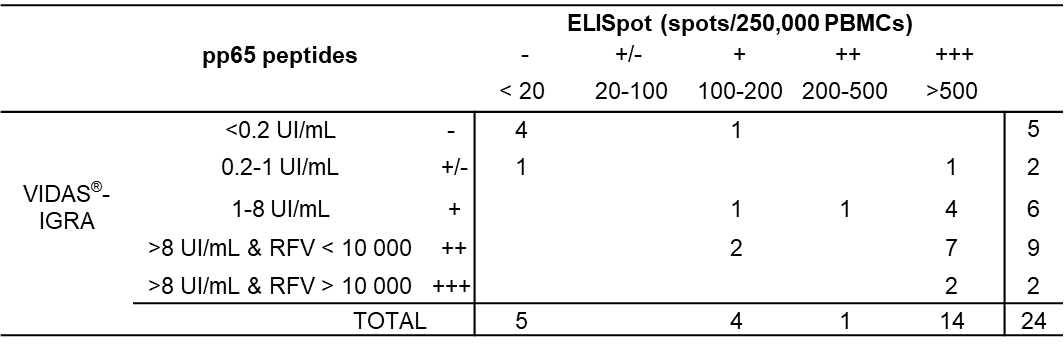


a


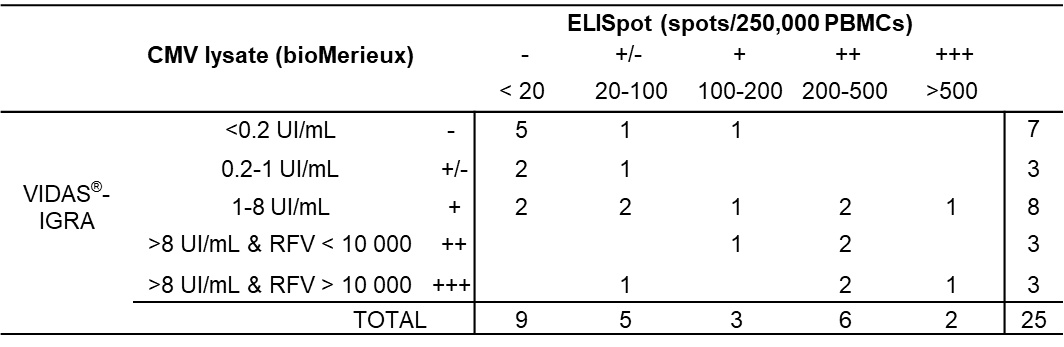


b

Result circled in red correspond to patients with negative CMV serology. RFV: fluorescence relative value

Supplemental Figure 2: Correlation of positive VIDAS^®^-IGRA results (relative fluorescence value (RFV) > 120) after activation of T-cells with pp65 peptide (JPT) or with pp65-IE-1 peptides pool (JPT). High Spearman’s correlation, ρ = 0.78.


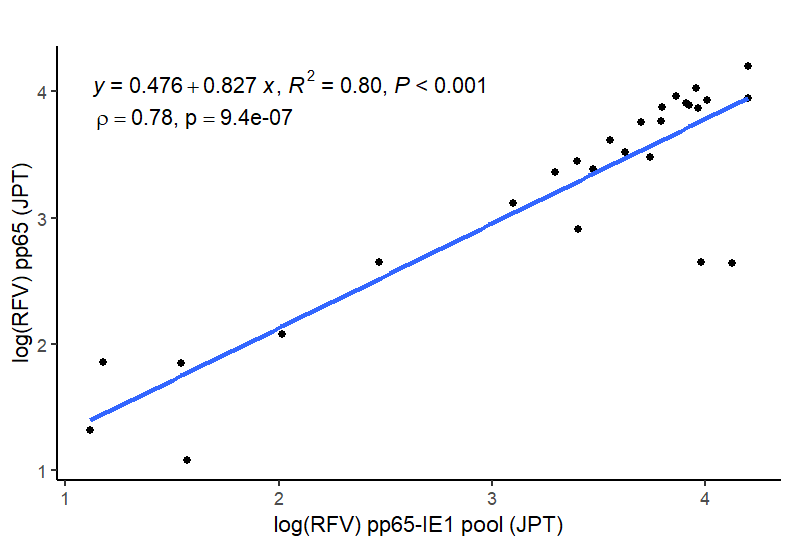


Supplemental Figure 3: Correlation of positive ELISpot results (> 20 spots/250,000 PBMCs) after activation of T-cells with pp65 peptide (JPT) or with pp65-IE-1 peptides pool (JPT). High Spearman’s correlation, ρ = 0.77.


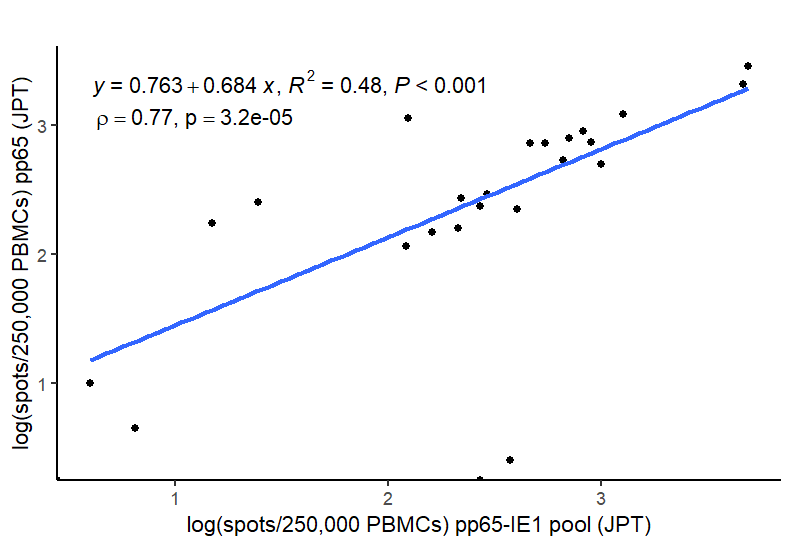

Supplement: Supplemental material — Fig. S1 to S3; Table S1. [file spectrum.02289-25-s0001.docx]
